# Cerebellar Cortex Granular Layer Interneurons in the Macaque Monkey Are Functionally Driven by Mossy Fiber Pathways through Net Excitation or Inhibition

**DOI:** 10.1371/journal.pone.0082239

**Published:** 2013-12-20

**Authors:** Jean Laurens, Shane A. Heiney, Gyutae Kim, Pablo M. Blazquez

**Affiliations:** 1 Department of Otolaryngology, Washington University School of Medicine, Saint Louis, Missouri, United States of America; 2 Department of Psychology, University of Pennsylvania, Philadelphia, Pennsylvania, United States of America; The Research Center of Neurobiology-Neurophysiology of Marseille, France

## Abstract

The granular layer is the input layer of the cerebellar cortex. It receives information through mossy fibers, which contact local granular layer interneurons (GLIs) and granular layer output neurons (granule cells). GLIs provide one of the first signal processing stages in the cerebellar cortex by exciting or inhibiting granule cells. Despite the importance of this early processing stage for later cerebellar computations, the responses of GLIs and the functional connections of mossy fibers with GLIs in awake animals are poorly understood. Here, we recorded GLIs and mossy fibers in the macaque ventral-paraflocculus (VPFL) during oculomotor tasks, providing the first full inventory of GLI responses in the VPFL of awake primates. We found that while mossy fiber responses are characterized by a linear monotonic relationship between firing rate and eye position, GLIs show complex response profiles characterized by “eye position fields” and single or double directional tunings. For the majority of GLIs, prominent features of their responses can be explained by assuming that a single GLI receives inputs from mossy fibers with similar or opposite directional preferences, and that these mossy fiber inputs influence GLI discharge through net excitatory or inhibitory pathways. Importantly, GLIs receiving mossy fiber inputs through these putative excitatory and inhibitory pathways show different firing properties, suggesting that they indeed correspond to two distinct classes of interneurons. We propose a new interpretation of the information flow through the cerebellar cortex granular layer, in which mossy fiber input patterns drive the responses of GLIs not only through excitatory but also through net inhibitory pathways, and that excited and inhibited GLIs can be identified based on their responses and their intrinsic properties.

## Introduction

**Figure 1:**
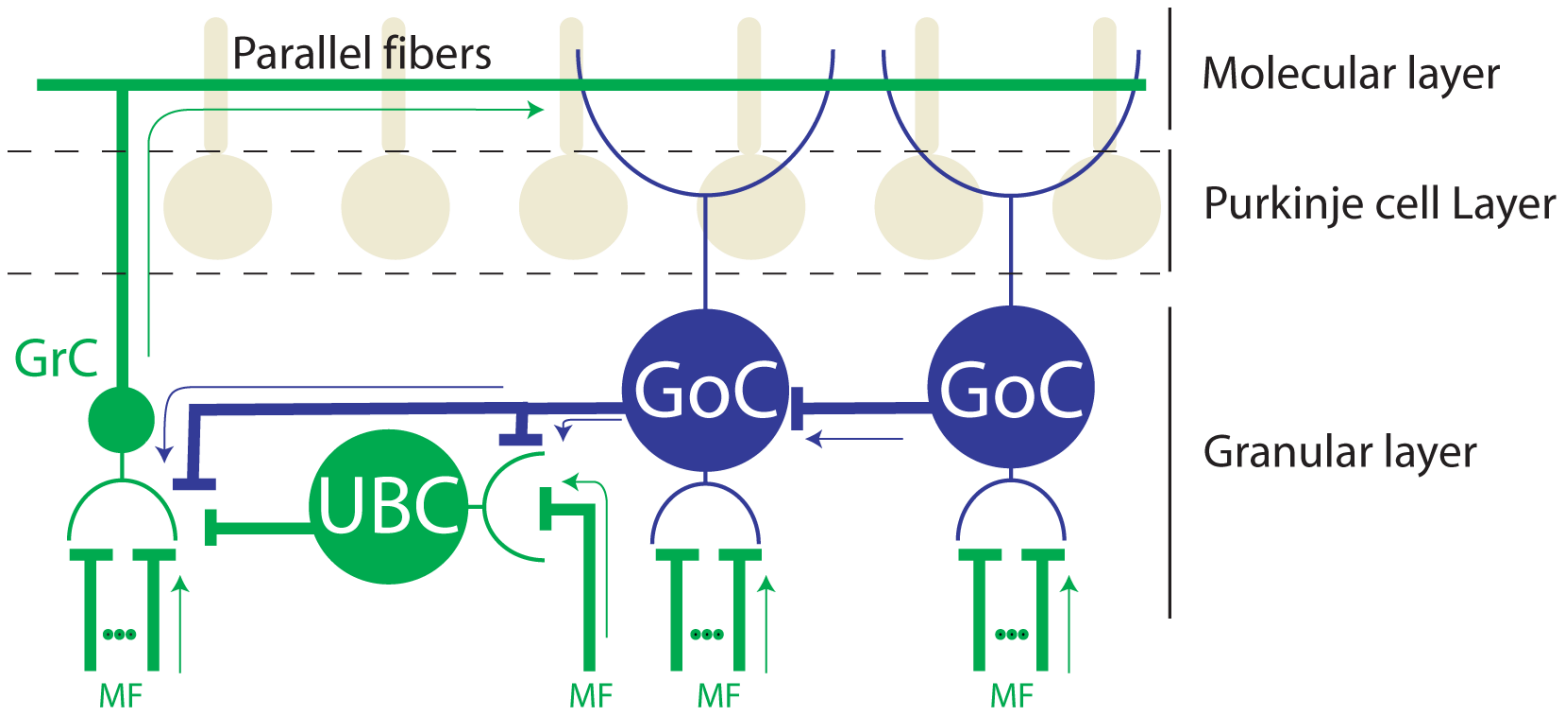
Schematic diagram illustrating direct and indirect pathways connecting mossy fibers (MF) to GLIs. Arrows indicate the directionality of the signal flow. Granule cells (GrC) receive glutamategic inputs from MFs and UBCs, and glycinergic/GABAergic inputs from Golgi cells (GoC). Unipolar Brush cells (UBC) receive a single mossy fiber input and glycinergic/GABAergic inputs from nearby Golgi cells. UBCs in turn establish glutamatergic synapses with granule cells and other UBCs. Golgi cells receive glutamatergic inputs from mossy fibers and parallel fibers, and glycinergic/GABAergic inputs from other Golgi cells. For simplicity we show only the classical major connections and interneurons of the granular layer. We also separate them between glutamatergic pathways (green) and glycinergic/GABAergic pathways (blue), although we acknowledge that other neurotransmitters, pathways, and interneurons exist. Axons and dendrites are represented by thick and thin traces respectively.

Influential theories of cerebellar cortex posit a fundamental role of granular layer local circuit neurons, collectively called Granular Layer Interneurons (GLIs), in the computations carried out by the cerebellar cortex [1]–[3]. Indeed, these interneurons perform the first transformations of the input signals arriving in the cerebellar cortex. The input signals themselves are carried exclusively by mossy fibers, which convey the entirety of the extrinsic information arriving in the granular layer; thus all responses of granular layer cells are driven by a combination of extrinsic mossy fiber signals and intrinsic local circuit computations. Of the GLIs contributing to these local circuit computations there are two different classes of neuron that are thought to play the largest roles. Unipolar Brush cells (UBCs) are glutamatergic interneurons thought to receive direct mossy fiber excitation, which has led to the hypothesis that they play a role in amplifying mossy fiber signals (Figure 1) [3]. Golgi cells are inhibitory interneurons [4] thought to sample mossy fiber activity directly through their descending dendrites and indirectly via parallel fibers through their ascending dendrites (Figure 1), which allows them to provide both feedforward and feedback inhibition of granule cells and other GLIs.

Despite the accumulating evidence pointing to a key role of GLIs in granular layer processing we still do not how they operate. This is due in part to the difficulty of recording and identifying interneurons in awake behaving animals [5]–[7], which is critical in order to examine how the interneurons process information during cerebellar dependent behaviors. We recently analyzed data from paired simultaneous recordings of GLIs and mossy fibers in the squirrel monkey and found that the firing rates of these two types of units were generally negatively correlated. This observation led us to suggest that GLIs are functionally driven by a small number of independent mossy fiber-derived inputs through net inhibition [7]. Our previous study and one performed by Prsa and colleagues [6] in the oculomotor vermis are the only major studies that have looked specifically at the responses of GLIs in the awake primate. However, neither prior study was able to characterize the responses of all GLIs, probably because GLIs are made up of a hetereogeneous population with diverse response properties.

Here we investigate mossy fiber and GLI responses in behaving macaques and provide the most detailed characterization to date of the responses of GLIs in the ventral paraflocculus (VPFL) of the awake primate. Our data suggest that GLIs can be organized into 5 discrete groups based on their response properties, but model fittings show that these groups share a common organizational principle. Namely, the seemingly complex discharge patterns of all groups of GLIs can be traced back to the response profiles observed in mossy fibers, with some GLIs reflecting a net excitatory effect of the mossy fiber pathway and others reflecting a net inhibitory effect.

## Materials and Methods

### Animal Preparation

We used three adult male rhesus macaques. These animals were not sacrificed and are still being used for other experiments. Animals were implanted with a head post for head restraint and a scleral search coil to monitor eye position. After a month of postsurgical recovery animals were trained in oculomotor tasks. Water intake was restricted to the experimental room during our training experiments (5 days per week), where animals received water until satiated once a day. Two days a week, usually weekends, animals received water in their cages (minimum of 35 ml/kg/day). Animals were provided with fruits and vegetables daily after experimental sessions and during weekends. After animal training was completed, animals underwent a second surgical procedure where they were implanted with a recording chamber aimed to the left floccular complex [8]. Surgeries were performed aseptically under 1–2% isoflurane anesthesia.

### Experimental setup

During recordings, animals were comfortably seated in a primate chair with their head fixed to the chair by a custom-made head post holder. The primate chair was mounted atop a rotating table that was used for earth vertical axis vestibular stimulation. Visual stimuli were delivered by a laser projection system that back-projected a red laser onto a tangential screen (93×93 cm) placed 50 centimeters in front of the animal. Eye movements were measured using an earth fixed coil system (CNC Engineering, Seattle, WA) and a reference coil mounted on top of the animal's head. A Power 1401 device and Spike 2 software (Cambridge Electronic Design, UK) were used for stimulus delivery and data acquisition. Eye, laser, and table position were digitized and acquired at 500 Hz, and raw neuronal activity at 40 KHz. Rightward and upward positions of the laser and eye were considered positive. Electrode microdrive displacement was recorded at 1 µm resolution.

### Behavioral protocols

We used standard positive reinforcement methods to train animals to follow visual targets on the screen [9]. We used four different tasks to test the neuronal responses: 1)“Pursuit”, which consisted of a sinusoidally moving laser (0.4 Hz and 10 deg amplitude) around the center of the screen. This task was used to test if the recorded neuron had eye related information. 2) “Off-center fixation”, which consisted of initial fixation in the center of the screen (1–1.5 s) followed by displacement of the target to a new location (5, 10, 15, 20 deg right, left, up or down from the center fixation) where the animal was required to maintain fixation for a random duration between 1–1.5 s. We used this task to calculate the relationship between neuronal firing rate and eye position for the main results of this study (see ‘data analysis’ below). 3) “Sequential saccades”, where animals were required to fixate a laser at an eccentric location (i.e. 20 deg or 15 deg away from center fixation) and follow it using a sequence of 5 deg saccades and subsequent fixation (1–2 s) to the other side of the projection screen. This task was used to confirm the presence of floor and ceiling effects in the neuronal response of some GLIs, as is predicted by their neuronal response during the “off-center fixation task”. 4) “VOR cancellation”, where the chair and the laser moved together sinusoidally at 0.4 Hz and 10 deg amplitude. Because our rotating chair system is fixed to an earth vertical rotational axis we were only able to deliver horizontal VOR cancellation.

### Neuronal recordings

Neuronal activity was recorded using high impedance tungsten microelectrodes (4–10 MOhms, FHC, Bowdoin, ME), amplified and bandpass filtered at 0.1–8 KHz (BAK Electronics, Stanford, FL). Single unit activity was sorted offline by a custom spike sorting script based on spike amplitude, peak derivative and principal component analysis, followed by manual inspection (Matlab, Mathworks, Natick, MA). Occasionally, we recorded a GLI and a mossy fiber simultaneously on the same electrode that could later be successfully sorted and separated as individual units using their spike shape. Neuronal recordings were aimed to the left floccular complex, specifically to the ventral paraflocculus (VPFL) [8]. The VPFL was identified by its characteristic strong saccade related activity, most prominent in the granular layer. We identified the three layers of the cerebellar cortex using standard criteria: i) the molecular layer was identified by the presence of complex spikes and the absence of large units, ii) the Purkinje cell layer was identified by the presence of Purkinje cells, identifiable by their simple spike pause (10 ms or more) following complex spikes, iii) the granular layer was identified based on its characteristic hashing activity, the absence of complex spikes and the presence of sparsely distributed neurons and mossy fibers (see also Results section and [7] for criteria to identify interneurons and mossy fibers). Additionally, three important features of our experimental methods helped us to confidently identify the three layers of the cerebellar cortex. 1) We aimed our recordings to lobules V–VIII of the floccular complex, which are stacked in a pancake-like manner in the coronal plane [8], [10]. This anatomical arrangement makes for an easy online identification of the three layers of the cortex. For example, most commonly we found blocks of the following: molecular layer followed by Purkinje cell layer, followed by granular layer, followed by silence (white matter), followed by granular layer, followed by Purkinje cell layer and followed by molecular layer (see Figure S1). 2) We saved the entire recording session (usually between 2–3 hours) as a single continuous data file that contained the behavior, the neuronal activity and the electrode depth (microdrive position). Thus, we could replay the entire recording session, or part of it, offline as many times as necessary to confidently assign each recorded cell to a cerebellar cortex layer. 3) Units in which the location was uncertain were removed from our data set. Because the spiking activity in the molecular, Purkinje cell, and granular layer are very easy to distinguish (see Movie 1), and because the pancake-like arrangement was encountered systematically, we are certain that all the neurons presented here were recorded in the granular layer.

### Data analysis

Data were imported into Matlab for offline analysis. Saccades were detected automatically using a 20 deg/s velocity threshold. Neuronal activity was used to calculate the average firing rate, the coefficient of variation of the logarithmic distribution of ISIs in ms (CVlog), the median interspike interval, the median CV_2_ (CV_2_ = 2 |ISI_n+1_-ISI_n_|/(ISI_n+1_+ISI_n_)) and the fifth percentile of the ISI distribution. These calculations were used to classify interneurons according to the criteria developed by Ruigrok and colleagues and Dijck and colleagues [11], [12], but as we will show below the classification was unreliable.

Neuronal responses to sinusoidal stimulation (VOR cancellation and pursuit) were described by the best fitting sinusoidal equation to the average neuronal response over at least 4 sinusoidal cycles. We used the off-center fixation task to study the relationship between neuronal firing rate and eye position. This task was preferable to spontaneous fixation or sequential saccades because GLI firing following a saccade often depends on the recent history of the eye position before the saccade [9]. The off-center fixation task controls for these possible history effects by always imposing the same starting eye position. The neuronal response was quantified as the average neuronal firing rate from 200 to 700 ms following peak saccade velocity, and each trial was counted as a single data point.

We estimated online whether cells responded preferentially to horizontal or vertical eye movements by performing a few off-center fixation trials along both orientations. Then we performed the complete off-center fixation task along the preferred orientation. The online estimation was later verified offline by comparing the variance in neuronal response during fixation trials along the estimated preferred (V) and non-preferred (V′) orientation. We considered that cells did indeed respond preferentially in the estimated orientation if V>V′*2, and that it responded approximately equally in both orientations if 0.5*V′<V<V′*2. There were no cells for which V<V′*0.5, i.e. for which we misclassified the preferred orientation online.

### Attempt to identify interneurons and mossy fibers

Unit classification was performed offline using spike shape and the spontaneous firing properties of the neurons. Briefly, mossy fibers were easily identified based on their sharp spike profile, consisting mainly of waveforms with one dominant phase, but occasionally triphasic. They are difficult to maintain in isolation and once their activity is lost it cannot be recovered by moving the electrode. On the other hand, GLIs show wider spikes, like those of Purkinje cells, with clear positive and negative phases in their waveforms. They can be recorded for tens of microns of electrode movement and can often be maintained well isolated for more than one hour.

We attempted to classify GLIs using the identification method recently proposed by Ruigrok and colleagues ([11], Figure S2) and Van Dijck G et al. [12] in the anesthetized rodent but, as we will explain in the Discussion, our evidence suggests that the classification methods developed for the anesthetized rodent cannot be reliably applied to our recordings in the awake macaque. Hence we have chosen a conservative approach: to present our data without pre-assigning GLIs to UBCs or Golgi cells.

### Classification of the response profiles of GLIs

Our fitting method evaluates the relationship between mean neuronal discharge and mean eye position following a saccadic eye movement (200–700 ms). We used linear or piecewise linear functions (see File S1) that can account for the response characteristics observed in our GLI population. The simplest function, F_1_, is a linear function that can contain a rectification at zero firing rate to account for the recruitment threshold of the neuron. Our second function, F_2_, contains a constant value above zero plus the element F_1_ multiplied by a coefficient (k). The coefficient k could take a value of 1 or −1. A value of 1 would generate a response profile similar to F_1_, but with a rectification above zero. A value of −1 would generate the mirror image of F_1_. Our third function, F_3_, is a piecewise linear function with two different slopes.

For statistical purposes, we used another function F_0_(x) = FR_0_, which assumes that the neuron doesn't respond to eye movement. We computed the sum of squares residual of the fits performed with F_0_,…,F_3_ and the variance accounted for (VAF). The best fitting function was selected by using a sequential F-test (see File S1). We considered that the increase of VAF from one method to another was significant if the associated p-value was less than 0.05 and the increase of VAF was higher than 2%. If no fitting method was found to be significantly better than F_0_, the cell was considered non-responsive and excluded from subsequent analysis.

### Ethical approval

All procedures regarding animal experimentation conformed to NIH guidelines found in the Guide for the Care and Use of Laboratory Animals and were approved by the Washington University Institutional Animal Care and Use Committee (protocol number 20120155). Washington University is well recognized for its strong effort to maintain a healthy environment for our experimental animals. Monkeys are housed in large cages that allow them to exercise and are frequently placed in larger play-cages. Animals are fed twice daily with food pellets, supplemented with a variety of fruits, vegetables and multivitamin complexes. Steps taken to alleviate animal suffering are set to the highest standards in Washington University. Thus, animals are monitored at least twice daily by veterinary personnel and at least twice daily by lab personnel (once daily during weekends). Animal rooms are set to a constant temperature of 23.5 deg Celsius with a regular 12 hour day/night cycle. The body weight of each animal is monitored at least once a week. Additionally, lab members and veterinary personnel monitor daily the motor behavior of the animals as well as their balance, posture and interaction with neighbors. Changes in animal behavior or body weight are taken as signs of distress or discomfort and are immediately reported to the veterinary personnel. In such cases we stop experimentation until animal behavior or body weight returns to normal. A strong environmental enrichment program is active in Washington University to provide toys and to pair animals for social interaction. All issues regarding animal care and welfare are under the direct supervision of the Washington University Medical School veterinary personnel.

## Results

We recorded the activity of 34 eye-related GLIs and 30 eye-related mossy fibers in the VPFL during our off-center fixation task. Twenty-nine out of 34 GLIs and 27/30 mossy fibers responded preferentially to eye movements along one orientation (horizontal or vertical); the remaining 5 GLIs and 3 mossy fibers had comparable sensitivities in both orientations.

### Mossy fiber responses

**Figure 2:**
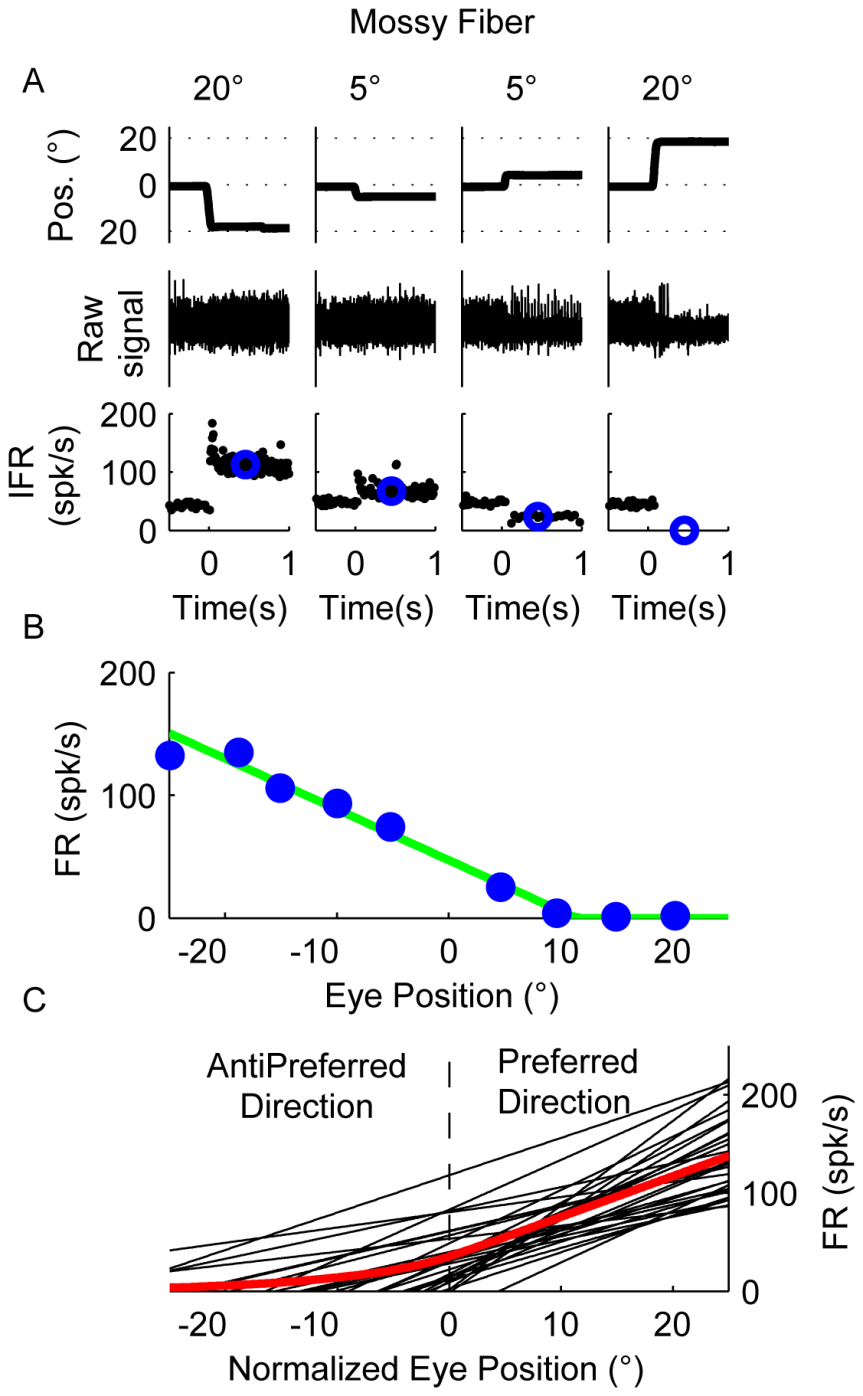
Responses of mossy fibers. (**A**) Raw data showing the response of an example mossy fiber to 5 deg and 20 deg leftward and rightward off-center fixation trials. The upper row shows horizontal eye position (negative values for leftward eye position and positive for rightward eye position), the middle raw spike data, and the bottom row instantaneous firing rate (IFR, black dots). Blue circles in the lower row represent the average firing rate following each saccade (200–700 ms). (**B**) Relationship between average firing rate and eye position using data from trials like those shown in A. (**C**) Best fitting line for each mossy fiber that could be well fit with F_1_, showing the relationship between eye position and firing rate. The eye position has been normalized to display the preferred direction of the neuron as positive. The average response profile across all mossy fibers is shown in red.

Eye movement related mossy fibers modulate their firing rate during sinusoidal pursuit with lesser or no response during VOR cancellation (median modulation of 50 spk/s and 12 spk/s during pursuit and VOR cancellation respectively, n = 6). Figure 2 shows the response of a typical mossy fiber during a series of off-center fixation trials. The mean firing rate increased linearly with leftward fixations as long as the eye remained to the left of +10 deg, however for eye positions to the right of +10 deg the firing rate was zero because of the recruitment threshold of the neuron ([5], Figure 2A and B). This response profile could be represented by the green line shown in Figure 2B, which corresponds to a linear regression line plus a rectification to prevent the firing rate from falling below zero spk/s for eye positions to the right of +10 deg.

All but one mossy fiber (29/30) increased their response linearly with eye position (Figure 2C). Most of them (25/29) showed a piecewise linear response shape characterized by a region of zero firing rate until an eye position recruitment threshold is met followed by a linear increase in firing rate with fixation toward the neuron's preferred direction, like the example neuron in Figure 2B. Mossy fibers could have ipsilateral (i.e. leftward, 41%, 12/29), contralateral (i.e. rightward, 24%, 7/29), upward (28%, 8/9) or downward (7%, 2/29) preferred directions. Because most mossy fibers had a recruitment threshold, the response curve across our sample of fibers exhibited a clear inflection point.

### GLI responses

In line with our previous work, we observed that GLIs responded to eye movements but not to head movements [7]. We measured the responses to horizontal smooth pursuit and VOR cancellation (0.4 Hz, 10 deg amplitude) in 15 GLIs that responded preferentially to horizontal eye movements. As expected, the median modulation during smooth pursuit (8.7 spk/s) was significantly larger than during VOR cancellation (1.8 spk/s, p = 0.01, Wilcoxon Rank Sum test) suggesting that these GLIs contain little or no head velocity information.

The responses of 34 GLIs were studied during the off-center fixation task in their preferred orientation. We found that our GLI population can be divided into 5 groups based on their characteristic response patterns during the off center fixation task (Table 1, first column). For each of the five groups we propose fitting methods to quantitatively describe the response profiles observed in the GLIs.

**Table 1:** Types of GLIs categorized based on their response profile during the “Off-center fixation” task; mossy fibers (MF) are shown in the bottom row for comparison.

GLI group	N	Best fitting function	Interpretation
I	10	F_1_	Undecided
II	6	F_2_ (k = 1)	Excited
III	8	F_2_ (k = −1)	Inhibited
IV	6	F_3_ (s_1_ & s_2_>0)	Excited
V	4	F_3_ (s_1_ & s_2_>0)	Inhibited
MF	29	F_1_	

The first column indicates the GLI type (last row been mossy fibers [MF]). The second column indicates the number of unit recorded. The third column shows the chosen fitting functions F_1_- F_3_. The fourth column the interpretation, based on the model fit, of the net pathways connecting mossy fibers to GLIs (excitatory, inhibitory, or undecided).

**Figure 3:**
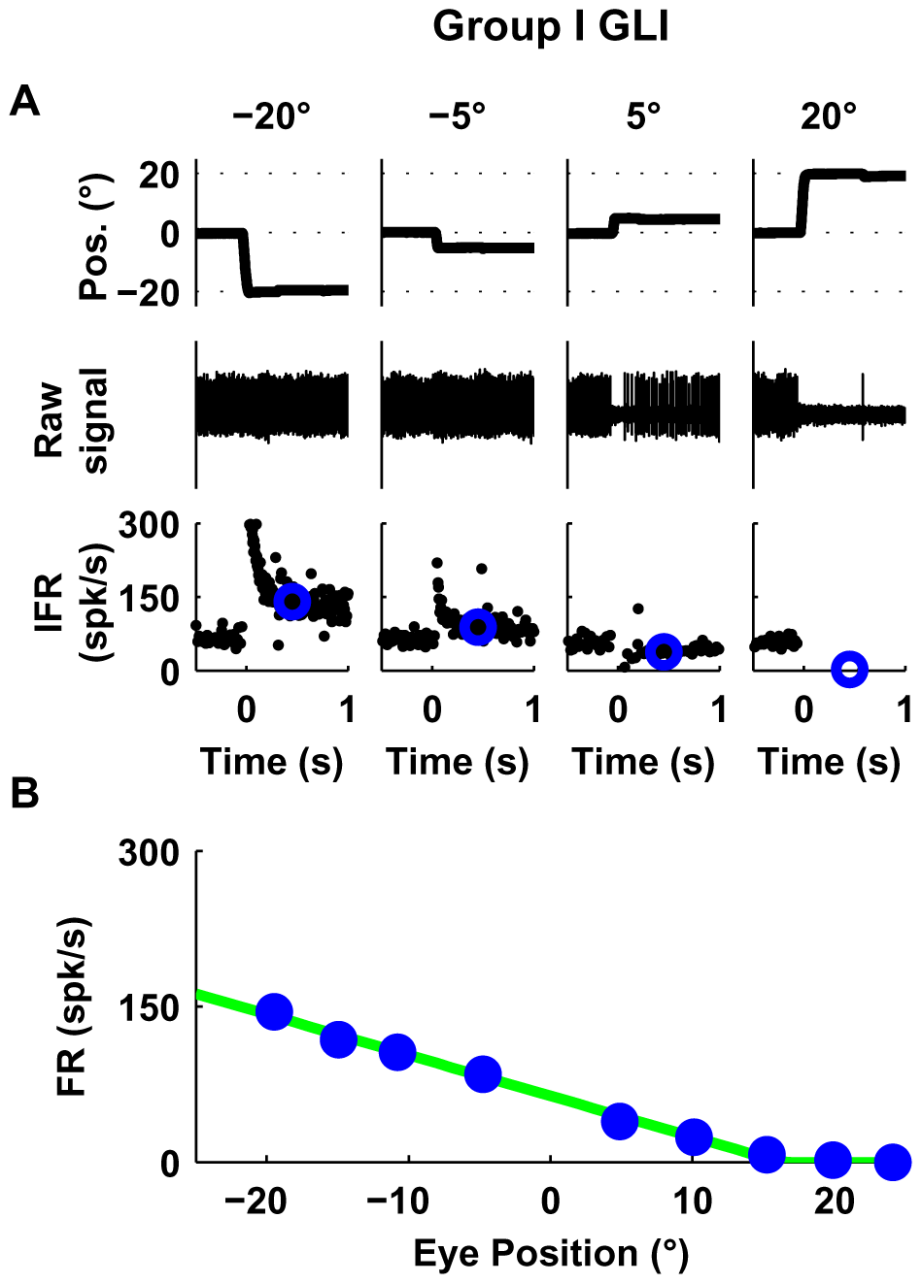
Response of one GLI with a linear response profile (Group I). (**A**) The upper row shows horizontal eye position, the middle raw spike data, and the bottom row instantaneous firing rate (IFR). Blue circles in the lower row represent the average firing rate following each saccade (200–700 ms). (**B**) Relationship between average firing rate and eye position using data from trials like those shown in A. The response of this example GLI was closely fit by a linear function F_1_ (VAF = 99.6%).

**Figure 4:**
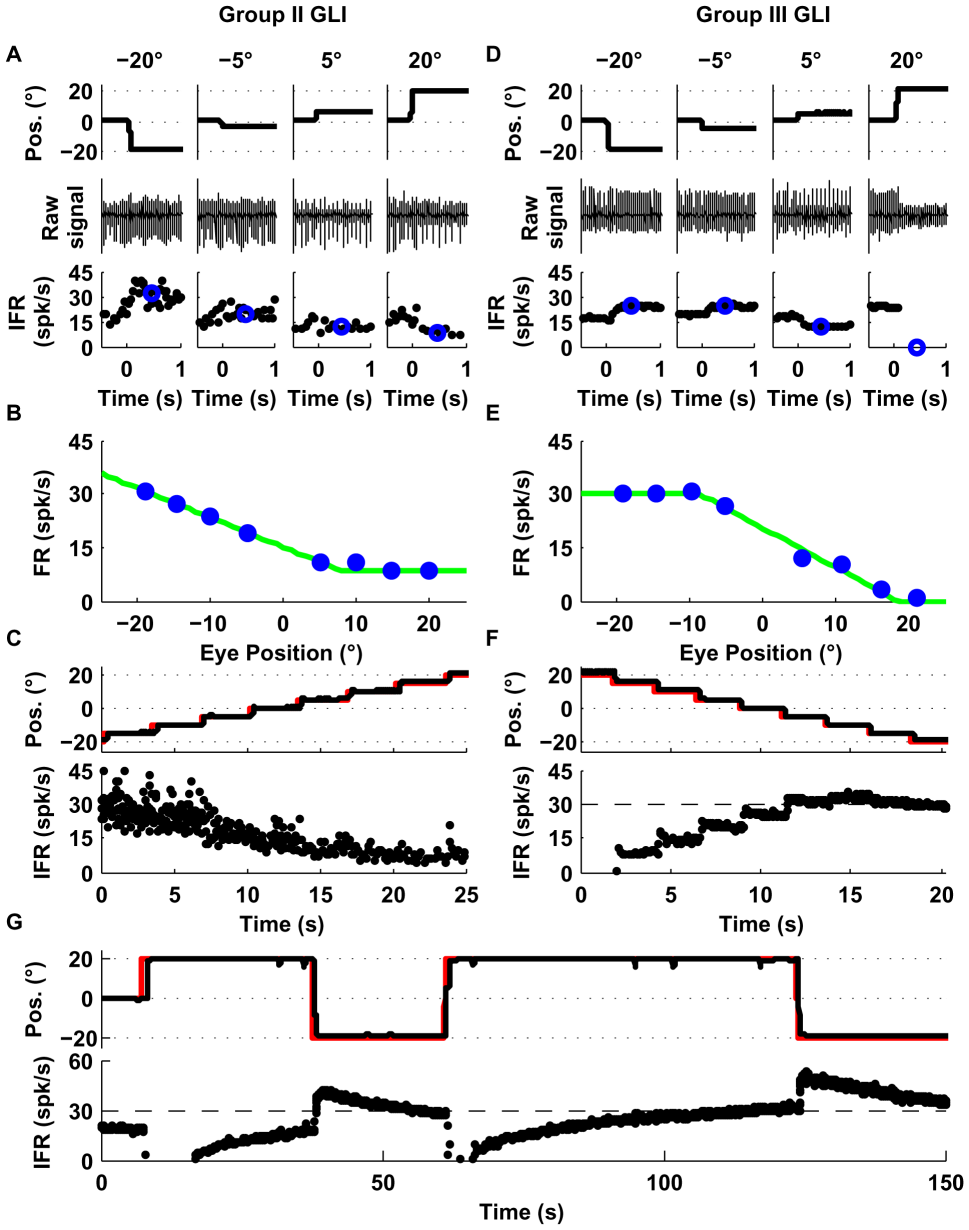
Responses of example Group II (A–C) and III (D-G) GLIs. (**A, D**) The upper row shows horizontal eye position, the middle raw spike data, and the bottom row instantaneous firing rate (IFR). Blue circles in the lower row represent the average firing rate following each saccade (200–700 ms). (**B, E**) Relationship between average firing rate and eye position using data from trials like those shown in A and D. Both cells were fit with the function F_2_. The corresponding fits are shown as green lines in B and E. (**C, F**) Response of the each example neuron during sequential saccades (5 deg). The upper row shows in black traces the eye position and in red traces the laser position; the lower row shows the instantaneous firing rate (IFR). (**G**) Response of the example Group III GLI shown in D–F during long eye fixations. The upper row shows in black traces the eye position and in red traces the laser position, the lower row shows the instantaneous firing rate (IFR).

**Figure 5:**
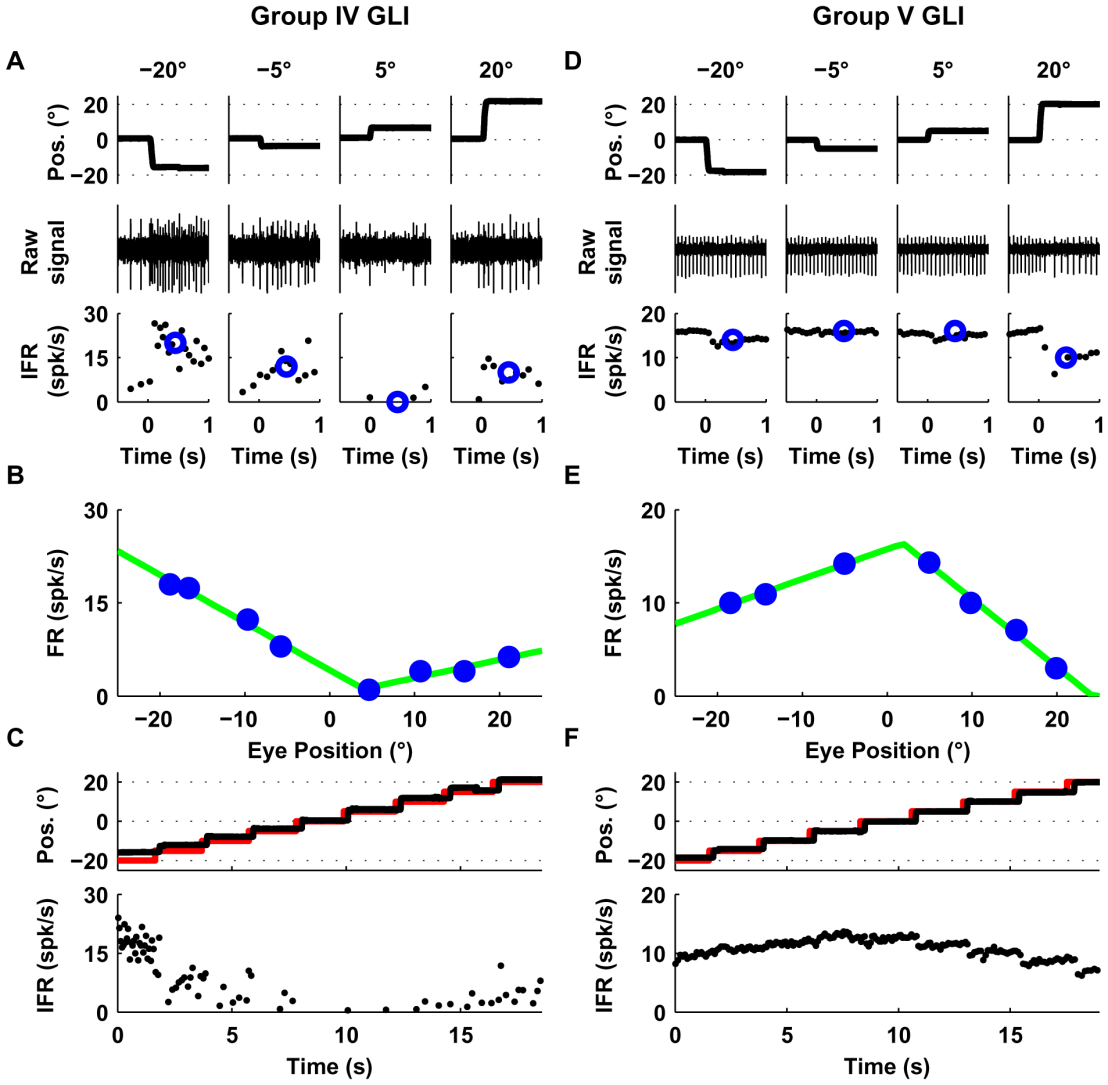
Responses of example Group IV (A–C) and V (D–F) GLIs. This figure uses the same layout as Figure 4A–F. (**A, D**) The upper row shows horizontal eye position, the middle raw spike data, and the bottom row instantaneous firing rate (IFR). Blue circles in the lower row represent the average firing rate following each saccade (200–700 ms). (**B, E**) Relationship between average firing rate and eye position using data from trials like those shown in A and D. Both cells were fit with the function F_3_. The corresponding fits are shown as green lines in B and E. (**C, F**) Response of the each example neuron during sequential saccades (5 deg). The upper row shows in black traces the eye position and in red traces the laser position; the lower row shows the instantaneous firing rate (IFR).

- Group I GLIs (10/34), showed a linear relationship between firing rate and eye position and their responses were best fit with an F_1_ function. The example neuron shown in Figure 3 increased its firing rate with leftward eye positions (Figure 3A) and showed a zero firing rate floor starting at around 15 deg, reminiscent of the mossy fiber recruitment threshold (Figure 3B, VAF obtained using F_1_ was 99.6%).
- Group II GLIs (6/34) showed similar response profiles as Group I neurons but had a non-zero floor effect (mean neuronal firing rate didn't decrease below a particular non-zero value). In the example shown in Figure 4A–C, the VAF obtained using F_2_ was higher (99%) than using F_1_ (94%), as shown by a sequential F-test (p = 0.005). The non-zero floor effect of group II neurons was apparent during sequential saccades as well (Figure 4C).
- Group III neurons (8/34) were characterized by a linear relationship between mean firing rate and eye position with a ceiling effect (mean neuronal firing rate didn't increase above a particular value). Some neurons in this group also showed a recruitment threshold. The response of the example neuron (Figure 4) was better fit by F_2_ than F_1_ (99% VAF vs. 95%, p = 0.008). The ceiling effect on the firing rate of this example neuron was also notable during sequential saccades (Figure 4F). Moreover, as shown in Figure 4G, the ceiling effect observed in Figure 4D–F was not due to firing rate saturation but to an eye position response field (i.e., range of eye positions in which the neuron shows eye related activity [7], which for the example neuron was 10 deg left to 20 deg right).
- Group IV (6/34) GLIs show a “V-shaped” response profile during the off-center fixation task (Figure 5A, B). The response profile of group IV neurons was best fit by a function that uses two lines with distinct slopes (F_3_), which provided a higher VAF than F_2_ (99% vs. 94%, p = 0.01). (Figure 5A–C).
- Group V (4/34) GLIs show an “inverted V-shaped” response profile during the off-center fixation task. This response mirrors that of Group IV GLIs. The response of the example neuron (Figure 5D–F) was better fit by F_3_ than F_2_ (99% VAF vs. 83%, p = 0.004).

In summary, we found that the majority (24/34) of GLIs exhibited non-linear (piecewise linear) response patterns that were not the result of a recruitment threshold. These patterns were easily observed online: Group II and III GLIs were clearly non-responsive when the eyes moved in one half of the visual field; Group IV GLIs increased their firing rate during off-center fixations in both directions whereas Group V decreased their firing rate during off-center fixations in both directions. At first glance, this diversity of responses may be confusing and hinder efforts to analyze the response of GLIs. However, we found that it could be easily understood and interpreted, and that it could in fact be used to draw hypotheses about the functional connectivity of these neurons with mossy fibers.

### An interpretation of GLI responses based on mossy fiber responses

**Figure 6:**
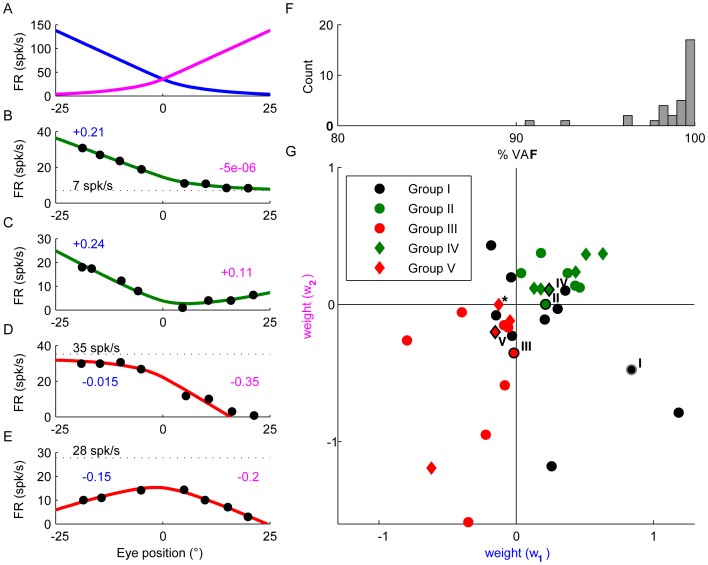
Modeling the responses of GLIs. (**A**) Average response profile of mossy fibers, identical to the red line in Figure 2C, but shown as two different tuning curves with opposite directional preferences for illustration of the fitting approach. (**B–E**) Model fits of the response profiles of the example Group II (B), IV (C), III (D) and V (E) GLIs. The weights w_1_ and w_2_ used for the fits are indicated in blue and magenta. The offsets FR_0_ are indicated by black broken lines (in C, the offset is negative). (**F**) Distribution of VAFs produced by the model across the population of GLIs. (**G**) Scatter plots showing the weights obtained by model fitting. The example Group I–V GLIs shown in Figure 3–5 are plotted with black (group II–V) and gray (group I) borders and with roman numbers to the left indicating the GLI group they belong to. The asterisk indicates a group V GLI with a w_2_/w_1_ ratio near zero (the response profile of this GLI is shown in Figure S3). This cell was atypical among group IV and V GLIs because, unlike the rest of group IV and V GLIs whose responses were best fit by two slopes of opposite sign (i.e., direction), the slopes of the two lines that best fit the response of this cell had the same direction.

We offer an interpretation of the response of GLIs based on the observation that the piecewise linear response profile of group I–III GLIs resembles the response profile of a typical mossy fiber (e.g. Figure 2B), and that the response profile of group IV and V resembles the combined response of two mossy fibers. We used the average mossy fiber response as a template to fit GLI responses because GLI responses may be influenced by the activity of many mossy fibers (Figure 1, and Figure 2C) [4]. Our interpretation can be mathematically represented by the equation:
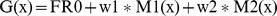
where M_1_(x) and M_2_(x) are the response profiles of mossy fibers (constructed from the average response of all mossy fibers) that respond in opposite directions (see Figure 6A), and w_1_ and w_2_ the weights. Note that the population response profile is not much different than the response of a typical mossy fiber with a recruitment threshold. Fitting using single mossy fibers instead of the average response profile indeed yielded the same final interpretation. This type of analysis is not shown here to facilitate the presentation of the data, but briefly, consisted of modeling GLI response profiles using one or two canonical mossy fiber responses where their sensitivity to eye position and the recruitment thresholds were set as free parameters. Our motivation for using the “pooled” M1 and M2 instead of just one or two single mossy fiber inputs to describe GLI responses, was to build a model that agrees with the known anatomical connections between mossy fibers and the canonical GLI (Golgi cells receives direct and indirect inputs from many mossy fibers).

The response of the example Group II GLI (Figure 4) could be explained by inputs from a single pool of mossy fibers with an ipsilateral preferred direction and a weight of +0.21 (Figure 6B). The weight of the other pool was close to zero. The V-shaped response profile of Group IV GLIs could be reproduced by inputs from both pools (Figure 6C). Interestingly, the response profile of Group III and Group V GLIs can be explained by assuming that the net weight of the pathways connecting these pools of mossy fibers to GLIs is negative (Figure 6D and E). The model generally fit the data very closely (see Figure 6B–E), with VAFs higher than 90% for all cells and equal to 98.5% on average (Figure 6F). In agreement with the examples shown in Figure 6B–E, we found that the net synaptic weights of mossy fiber pathways to Group II and IV GLIs were positive (Figure 6G), whereas those to Group III and V GLIs were negative. This suggests that “floor effects” and “V shaped” response profiles can be attributed to net excitatory pathways from mossy fibers, whereas “ceiling effects” and “inverted-V shaped” profiles can be attributed to net inhibitory pathways (see also Table 1, last two columns).

Group I GLIs were found distributed throughout the four quadrants of Figure 6G. One GLI fell in the same quadrant as group II and IV GLIs, suggesting that it received excitation from two pools of mossy fibers with opposite directional preference. Two GLIs fell in the same quadrant as group III and V GLIs, suggesting that they received inhibition from two pools of mossy fibers. The remaining 7 GLIs had fittings that were difficult to interpret. For instance 70% (5/7) had high recruitment thresholds, which increased the uncertainty of the fitting because there were fewer data points to fit, and 30% (2/7) showed monotonic responses throughout the entire eye movement field (+20 to −20 deg). These monotonic responses could be generated by combining an excitatory input from one pool of mossy fibers with one inhibitory input from the other pool of mossy fiber. However monotonic responses could also be generated through either excitation or inhibition if most mossy fibers influencing the GLI have similar response profiles and no recruitment thresholds. Overall, Group 1 GLIs cannot be classified with certainty as having net inhibitory or excitatory mossy fiber pathways, thus we have opted to label these neurons as undecided.

### Additional evidence supporting the separation of GLIs into different groups

**Figure 7:**
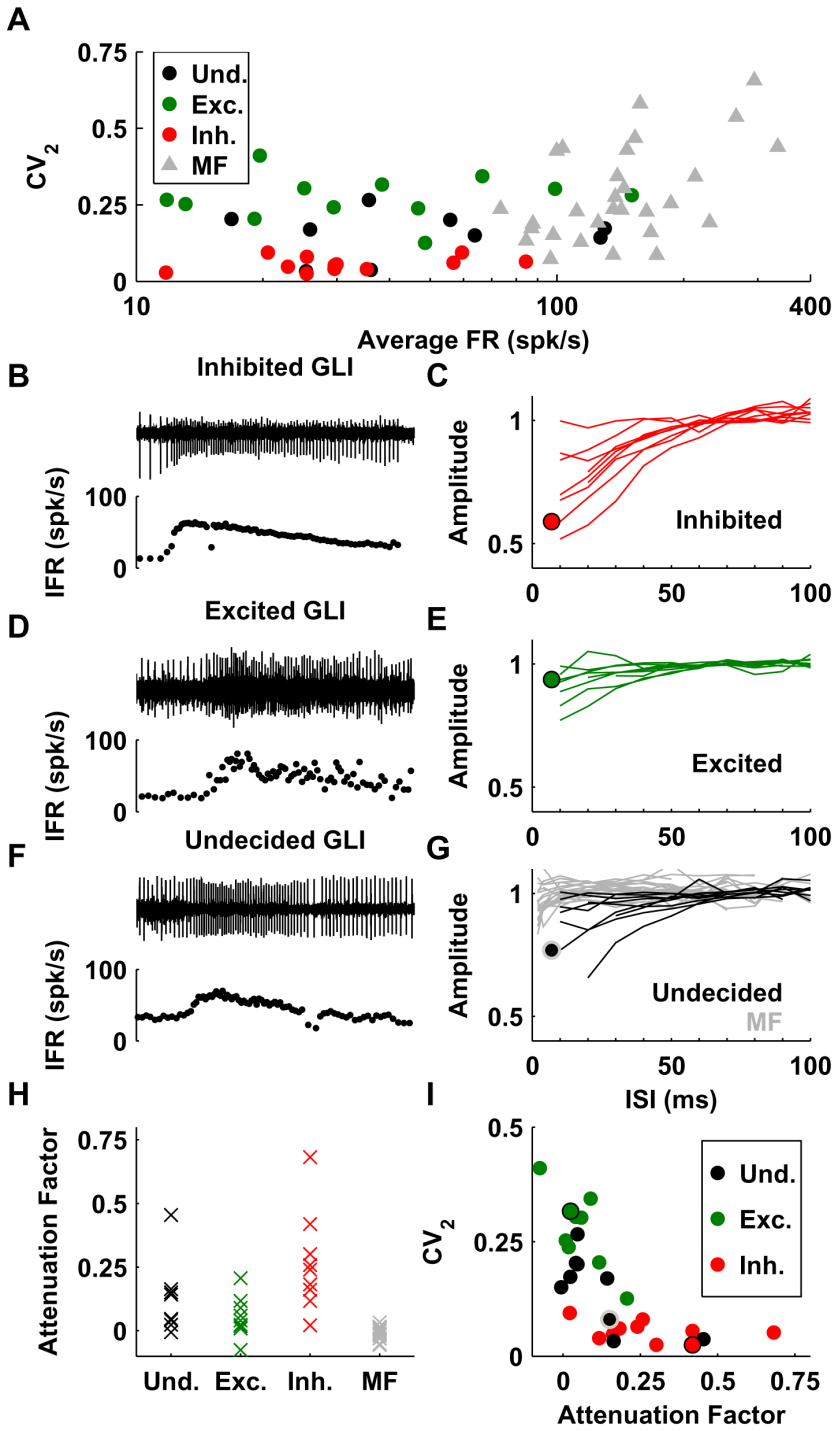
Firing properties of the GLIs and mossy fibers. (**A**) Average firing rate and CV_2_ of the various neuronal elements. GLIs (circles) are color-coded according to their response properties: ‘undecided’ in black, ‘excited’ in green, and ‘inhibited’ in red). (**B–G**) Attenuation of spike amplitude at high firing rates. Panels B, D and F show the raw trace (top) and IFR (bottom) of three example cells. Panel C, E and G show the normalized spike amplitude (1 corresponds to the median amplitude when ISI>50 ms) of GLIs and mossy fibers (G) as a function of interspike interval (ISI). The circles indicate the curves corresponding to the example cells in panels B, D and F. (**H**) Attenuation Factor (median spike amplitude when ISI>50 ms divided by the median spike amplitude when ISI<30 ms) of different GLI classes and of mossy fibers. (**I**) Relation between CV_2_ and Attenuation Factor in GLIs. The example cells in B, D and F are plotted with black (B and D) and gray (F) borders.

We can classify the five types of GLIs into three groups according to the predictions of our model fit: i) ‘excited’ GLIs, which are GLI types that according to our model receive excitatory inputs from mossy fiber pathways (Group II and IV, n = 12); ii) ‘inhibited’ GLIs, which according to our model receive inhibitory inputs (Group III and V, n = 12); and iii) ‘undecided’ GLIs, which are GLIs types that could be controlled by either excitatory or inhibitory pathways (Group I, n = 10). Interestingly, ‘inhibited’ GLIs have smaller median CV_2_ values than ‘excited’ GLIs (median: 0.05 vs. 0.27, Wilcoxon Rank Sum test p≪0.001), indicating that their discharges are more regular, whereas their firing rates are similar (median: 30 spk/s vs. 34 spk/s, p = 0.9). By comparison, mossy fibers have much larger firing rates than GLIs (median: 141 spk/s vs. 30 spk/s p≪0.001, Wilcoxon Rank Sum test) and higher median CV_2_ values (median 0.24 vs. 0.15, p = 0.001, Wilcoxon Rank Sum test) (Figure 7A).

We made a further observation, which is that the amplitude of the spikes recorded in some GLIs decreased when the firing rate increased (Figure 7B). Interestingly, this phenomenon could be used to draw a further distinction between ‘excited’ and ‘inhibited’ GLIs. We investigated this spike amplitude attenuation by plotting the normalized spike amplitude as a function of inter-spike interval (ISI) in 27 of the 34 GLIs for which we had enough data to compute spike amplitude at each firing frequency. Nine of these 27 GLIs were classified as ‘excited’ (Figure 7C), 9 as ‘inhibited’ (Figure 7E) and 9 as ‘undecided’ (all of which were Group I GLIs, Figure 7G). The attenuation of spike amplitude for low ISI (i.e. high firing rate) was apparent for the majority of ‘inhibited’ GLIs (Figure 7B, C). Remarkably, we found that ‘excited’ GLIs showed almost no attenuation in their spike amplitude (Figure 7D, E). When we examined the population of ‘undecided’ cells, we found that some of them exhibited spike attenuation (Figure 7F) whereas others did not. This is consistent with the notion that spike attenuation is characteristic of GLIs that receive inhibition from mossy fibers and that the population of ‘undecided’ GLIs is a mixture of cells receiving excitation or inhibition. We quantified the phenomenon of attenuation by computing an ‘Attenuation Factor’, defined as the median spike amplitude when ISI>50 ms divided by the median spike amplitude when ISI<30 ms (Figure 7H). In agreement with our observations, the attenuation factor was higher in ‘inhibited’ GLIs (median: 0.24, range: 0.02/0.7) than in ‘excited’ GLIs (median: 0.04, range: −0.08/0.2) (Wilcoxon Rank Sum test, p = 0.004). For comparison, we also investigated whether mossy fibers exhibit spike attenuation. We computed the spike amplitude curves in 20 mossy fibers; because mossy fibers can reach higher firing rates than GLIs, we were able to compute these curves for ISIs as low as 3–5 ms. We observed that mossy fibers presented only a slight attenuation at very high firing rate (ISI<5 ms, i.e. at firing rate of more than 200 Hz). Note that the attenuation factor was designed to quantify amplitude attenuation occurring at moderate firing rates in GLIs (>33 spk/s), and it is only weakly sensitive to the attenuation at high firing rates. As a result, the attenuation factor of mossy fibers was close to zero (median: 0.0006, range: −0.06/0.03). For comparison, the median attenuation factor of 10 Purkinje cells recorded in the same animals during similar protocols was 0.02 (range: −0.01/0.04). We also observed that the discharge regularity and attenuation factor were correlated: high attenuation factor is associated with low CV_2_ (Spearman's rank correlation: r = −0.73, p = 1.5*10^−5^) (Figure 7I). These observations suggest that ‘excited’ and ‘inhibited’ GLIs form two distinct populations.

An additional difference between ‘excited’ and ‘inhibited’ GLIs resides in their dynamic properties, which we quantify here as the ‘burst-tonic ratio’; maximum firing rate within the first 50 ms following saccade peak velocity versus maximum firing rate between 100 and 150 ms after saccade peak velocity. ‘Excited’ GLIs have a median burst tonic ratio of 1.4, while ‘inhibited’ GLIs have a median burst tonic ratio of 0.4. The differences in burst tonic ratio were significant (p≪0.001, Wilcoxon rank test). In comparison, mossy fibers have a median burst tonic ratio of 2.3.

Data collected from paired recordings of mossy fibers and GLIs further support the results of our model fittings. Out of 8 paired recordings of mossy fibers and GLIs, 4 pairs contained ‘excited’ GLIs and 3 pairs contained ‘inhibited’ GLIs. In support of our modeling results, mossy fibers recorded simultaneously with 3 of the ‘excited’ GLIs shared the same directional preference as their paired GLI, while the mossy fibers recorded simultaneously with 2 of the ‘inhibited’ GLIs showed the opposite directional preference of their paired GLI. The remaining 3 pairs contained mossy fibers and GLIs with unrelated responses.

## Discussion

Our study provides the best characterization to date of the responses of individual GLIs in the awake animal, and offers clues about the functional connectivity between mossy fibers and GLIs. We observed that the responses of many eye related GLIs in the VPFL exhibited floor or ceiling effects (Figure 4) as well as more complex response profiles (Figure 5), all of which could be explained by using a simple modeling approach. Our model fittings used mossy fiber and GLI responses to infer the number of distinct mossy fiber response profiles influencing the response of each GLI and the net effect (excitatory or inhibitory) of the pathways connecting mossy fibers to GLIs. Two predictions from our results are surprising: 1) The response profile of many GLIs can be explained by individual mossy fibers or mossy fiber pools with the same directional preference for eye movements (i.e., group II and III GLIs), and 2) Mossy fiber input pathways have a net inhibitory effect on some GLIs. In addition, the marked differences in the intrinsic properties (regularity and spike attenuation), and dynamic properties (burst-tonic ratio) of ‘net excited’ and ‘net inhibited’ GLIs suggest the existence of at least two distinct classes of GLIs in our population of recorded neurons. Our results in the awake animal find support in recent data obtained in anesthetized and *in vitro* studies [17]–[19], [25], and provide valuable knowledge to be incorporated into current theories of granular layer processing.

### Types of GLIs recorded

We assume that our data set does not contain granule cells because they are very small neurons that require high impedance electrodes so it is highly unlikely that they can be isolated using our metal microelectrodes [5]. However, our data set should contain GLIs known to exist in the vestibulo-cerebellum, such as Golgi cells and UBCs. No study to date has recorded these GLIs in awake behaving primates and identified them using postmortem histology. To our knowledge the only studies that have recorded these neurons and identified them postmortem were carried out in anesthetized animals [11], [12].

The identification method recently proposed by Ruigrok and colleagues in the anesthetized rodent ([11], Figure S2) does not appear to be well suited for our awake behaving monkey dataset because: 1) This classification method yields at least a 15% false negative rate for our GLIs: 5 GLIs were classified as molecular layer interneurons. 2) Only one of the 54 recorded GLIs (34 eye related and 10 not eye related) was classified as Golgi cells (one of the non eye related units), which is surprising since Golgi cells should be frequently isolated in the granular layer due to their large cell bodies. This suggests that the firing properties that Ruigrok and colleagues used for their classification of cerebellar cortical interneurons in anesthetized rodents cannot be reliably applied to GLIs in awake monkeys. We investigated the reason why Ruigrok's method produced this result and found that most neurons were classified as ‘unidentified’ or molecular layer interneurons because their CV2 was higher than 0.24 (not UBCs) and their ISI %5<0.02 (wrongly classified as molecular layer interneuron if CV2>0.24).

In a recent large-scale collaboration involving many labs and species, we used histologically identified interneurons in anesthetized mice, rats, and cats to investigate more flexible methods for classification of GLIs in both anesthetized and awake preparations [12]. Contrary to the criteria used by Ruigrok and colleagues [11], our classification method found that most of the GLIs included in the present work were Golgi cells. A caveat of the multi-species classification method is that the classifier was not trained for regions rich in UBCs (e.g. the vestibulo-cerebellum) and could thus potentially mistakenly classify UBCs as Golgi cells. However, a major finding of the cross-species comparison was that the regularity of Golgi cells tends to increase with increasing firing rates, which suggests that Golgi cells in awake preparations (in which firing rates are generally higher) could be misclassified as UBCs based on regularity criteria such as CV_2_. Hence, as happens for many other central nervous system neurons (see [13]), GLI firing properties, and classifications based on these properties, are likely to change with the recording site, animal species, animal state (anesthetized vs. awake), and influence of anesthetics.

In the following sections we describe our results without pre-assigning GLIs to UBCs or Golgi cells because in light of the discussion above we feel that the classification methods available today cannot reliably separate Golgi and UBCs recorded in the awake primate.

### Circuits and mechanisms underlying GLI responses

Based on the success of our fitting results, the simplest explanation for the GLI responses described here is that they are generated by the inputs from mossy fiber pathways interacting with the intrinsic properties of the neurons, such as their spontaneous firing. Mossy fiber responses alone can generate eye position response fields (range of eye positions where a GLI is responsive) and determine the directional preferences of GLIs. When the mossy fiber signals are combined, through excitation or inhibition, with the spontaneous firing pattern of the neuron, phenomena such as ‘floor’ and ‘ceiling’ effects can emerge. As shown in Figure 4G, such effects are not the result of an intrinsic firing rate saturation of these neurons but rather from a lack of responsiveness of the mossy fiber inputs for particular eye positions. Based on these ideas, we offer a simple and informative way of evaluating GLI responses. GLIs no doubt have a variety of additional intrinsic, synaptic, and network properties that confer other characteristics such as their long and short time constants [7], spike attenuation, and spike firing regularity, but our modeling results demonstrate that despite the apparent heterogeneity in these additional properties the responses of all GLIs can be predicted from a linear combination of mossy fiber inputs.

Our results suggest that some GLIs receive net excitation from mossy fibers and some net inhibition. This was supported by comparing the responses of GLIs and mossy fibers during the off-center fixation task, our paired recordings of GLIs and mossy fibers, and the separation of GLIs based on discharge properties (spike attenuation, CV_2_).

We previously reported in the squirrel monkey that the responses of many GLIs are anti-correlated with those of nearby mossy fibers, but we did not find strong evidence of correlated excitatory activity. This is probably because our previous data included only GLIs whose responses could be analyzed using the short intersaccadic fixation periods characteristic of squirrel monkeys [9]. Such an analysis is only possible with the more regular neurons, which have lower CV_2_ and correspond to GLIs classified here as being inhibited by mossy fibers. GLIs with V-shaped and inverted V-shaped response profiles would not have been detected in our previous study because the algorithm used in our previous report to calculate the eye position fields in the squirrel monkey by necessity assumed a single preferred direction.

Excitatory mossy fiber pathways have been shown to occur directly by glutamate-mediated excitation through AMPA and NMDA receptors, which are present in Golgi cells and UBCs, and indirectly, in the case of Golgi cells, through glutamate mediated excitation by parallel fibers [14]–[16] (Figure 1). However, the possibility that net mossy fiber activity inhibits GLIs has only recently been suggested [9], [18]. Based on the known anatomy and neurochemical make up of GLIs there are two possible mechanisms that can explain a net inhibitory nature of pathways from mossy fibers to GLIs. The first mechanism is feedforward inhibition through inhibitory interneurons and the second is mGluR2-mediated hyperpolarization.

The only known interneuron mediated inhibitory pathway from mossy fibers to GLIs (Golgi cells and UBCs) is through Golgi cells because GLIs do not receive inhibition from molecular layer interneurons [3], [19]. A single Golgi cell could potentially inhibit many UBCs through the large Golgi cell axonal arborization, but because Golgi cells tend to occupy non-overlapping zones it would be difficult for these cells to synaptically inhibit other Golgi cells [4]. Golgi cells could also inhibit other Golgi cells through gap junction coupling, but it has recently been shown that this form of inhibition is best suited for slower temporal signaling (e.g. low frequency oscillations) [20], and not for the well-timed pauses observed in GLIs [7], [21], [22]. Nonetheless, using the data presented here we can envision a Golgi cell that receives net excitation from eye related mossy fibers and inhibits UBCs and nearby Golgi cells, effectively inverting the mossy fiber input signal at the level of GLIs and generating GLI type III and V responses.

Recent data suggest that mGluR2 mediated hyperpolarization could powerfully inhibit GLIs as well. Both Golgi cells and UBCs comprise neurochemically heterogeneous populations that can express mGluR2 receptors [23], [24]. UBCs and Golgi cells lacking mGluR2 receptors could be excited by glutamate, while those with mGluR2 could be inhibited by glutamate. In fact, this exact phenomenon has recently been shown for UBCs [17]. Likewise, others have previously shown that glutamate release decreases the discharge of Golgi cells through activation of mGluR2 receptors [18], [25].

Regardless of the mechanism(s) responsible for the observed excitatory or inhibitory influences of mossy fibers on GLIs, in providing the first full inventory of GLI responses in the VPFL of awake primates and suggesting a simple circuit-level explanation for their response profiles, our results should help improve GLI identification methods and contribute to the development of more realistic models of cerebellar cortex processing.

## Supporting Information

Figure S1 **reconstruction of the recording sites during an experimental session.** This figure shows a single electrode track (i.e. the depth of the electrode tip relative to its position at the beginning of the experiment) as a function of time (the duration of the experiment was about 2h30’, i.e. 9000s). The neuronal elements recorded and identified during the experimental session are shown at their respective time and depth. These elements were classified as Purkinje cells (PC), mossy fibers (MF) and Granular Layer Interneurons (GLI). Sites where complex spikes were recorded in the absence of notable simple spike activity are labeled as CS (cyan). On the basis of these recordings, we reconstructed the sequence of Purkinje cell layers (PCL), molecular layers (ML) and granular layers (GL) encountered during the experimental session. The inset shows the typical spike profiles of various cell types. The spiking profile of mossy fibers and complex spikes are unique and allow a definite identification of the molecular and granular layers. This, in turns, allows identifying GLI with certainty.
(TIF)

Figure S2 **Classification of GLIs according to Ruigrok et al. (2011).** (**A–D**) Average firing rate (A), CV2 (B), fifth percentile interval of the ISI distribution (C) and median ISI (D) as a function of the CV of the logarithm of firing frequency. Black lines represent the decision boundaries of the classification method. Circles, triangle and stars represent cells classified as ‘Unidentified’, ‘UBC’ and ‘Basket or stellate cells’. Green, red and black symbols correspond to cells which we classified as ‘Excited’, ‘Inhibited’ or ‘Undecided’. Note that this classification method follows a decision tree (see Fig. 8 in [18]). As an additional test, we investigated whether the classification method proposed by Ruigrok and colleagues [18] is sensitive to the portion of data selected for neuronal identification. Specifically, if instead of using all the spikes obtained from a given neuron for its identification we used only a few consecutive seconds of data (portions of 30 s of data, using a moving window of 30 s) our GLI population could be sorted out differently. We computed the percentage of 30 s segments for which the classification was the same as when using the entire dataset (ID consistency). Nineteen out of 24 putative UBCs (E), 0 out 5 unidentified cells (F) and 2 out of 5 cells classified as molecular layer interneurons (G) had an ID consistency of more than 90%. Therefore, altogether, only 19/34 (56%) neurons were classified consistently as a given type of GLI. Overall, it appears that the spiking activity of granular layer interneurons recorded in the ventral paraflocculus of alert macaques differs substantially from the data recorded in anesthetized rodents [18]. As a consequence, the majority of cells which were firmly identified as GLIs were not classified as such by this method.
(TIF)

Figure S3 **Response profile of a GLI (classified as Group 5) that showed the same directional preference for eye movements when the eye was in the right and left eye position field.** All other group 4 and 5 GLIs have opposite directional preference, that is their response were best fit using two slopes of opposite sign.
(TIF)

File S1 (DOCX)

Movie S1 **spiking activity of different neuronal elements.** The movie shows the raw spike trace, instantaneous firing rate (lower traces), the eye position (upper left), and the spikes as they can be observed during a recording session. The soundtrack also reproduces the sound typically heard on an audio monitor. Recordings from 5 cells are shown: (1) a Purkinje cell (notice that complex spikes are clearly audible, and that the cell responded to ocular pursuit), (2) complex spikes recorded in the molecular layer, (3) a mossy fiber, which exhibited a characteristic sharp spike, high-pitch sound, high and regular firing rate and bursting response to saccades, (4) a GLI with a low and regular firing rate. Notice that the firing rate of this GLI decreased during downward eye movement but does not increased during upward eye movement: it followed a typical ‘I’ profile as that shown in Figure 4D–G. Notice also that a ‘hashing’ activity is audible in the background, which indicates the presence of nearby mossy fibers. (5) a GLI with a high and irregular firing rate. Notice that a nearby mossy fiber with hashing activity is also clearly audible, and that the firing rate of this GLI increased during eye movements to the left but does not decrease during eye movement to the right: it follows a typical ‘E’ profile as in Fig. 4A–C. Note: This movie plays well in our windows movie player (run in windows 7), but other movie players may have problems syncing the sound and image.
(MP4)
