# Supplementary figures and images for: Cerebellar Cortex Granular Layer Interneurons in the Macaque Monkey Are Functionally Driven by Mossy Fiber Pathways through Net Excitation or Inhibition
Source: PLoS One. 2013 Dec 20;8(12):e82239. doi: 10.1371/journal.pone.0082239 (PMC3869689; doi:10.1371/journal.pone.0082239)

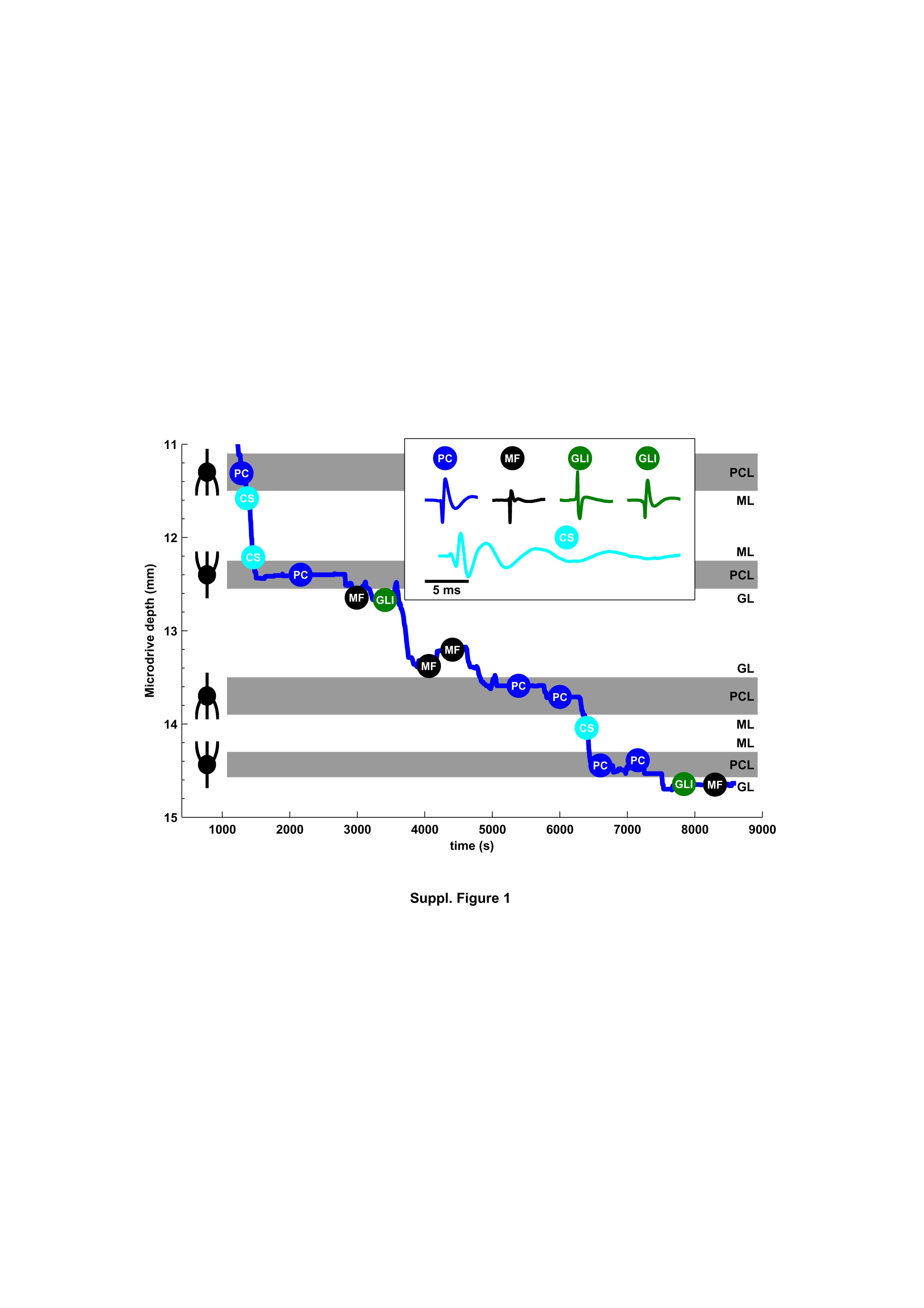

Supplement: Figure S1 — reconstruction of the recording sites during an experimental session. This figure shows a single electrode track (i.e. the depth of the electrode tip relative to its position at the beginning of the experiment) as a function of time (the duration of the experiment was about 2h30’, i.e. 9000s). The neuronal elements recorded and identified during the experimental session are shown at their respective time and depth. These elements were classified as Purkinje cells (PC), mossy fibers (MF) and Granular Layer Interneurons (GLI). Sites where complex spikes were recorded in the absence of notable simple spike activity are labeled as CS (cyan). On the basis of these recordings, we reconstructed the sequence of Purkinje cell layers (PCL), molecular layers (ML) and granular layers (GL) encountered during the experimental session. The inset shows the typical spike profiles of various cell types. The spiking profile of mossy fibers and complex spikes are unique and allow a definite identification of the molecular and granular layers. This, in turns, allows identifying GLI with certainty. (TIF) [file pone.0082239.s001.tif]

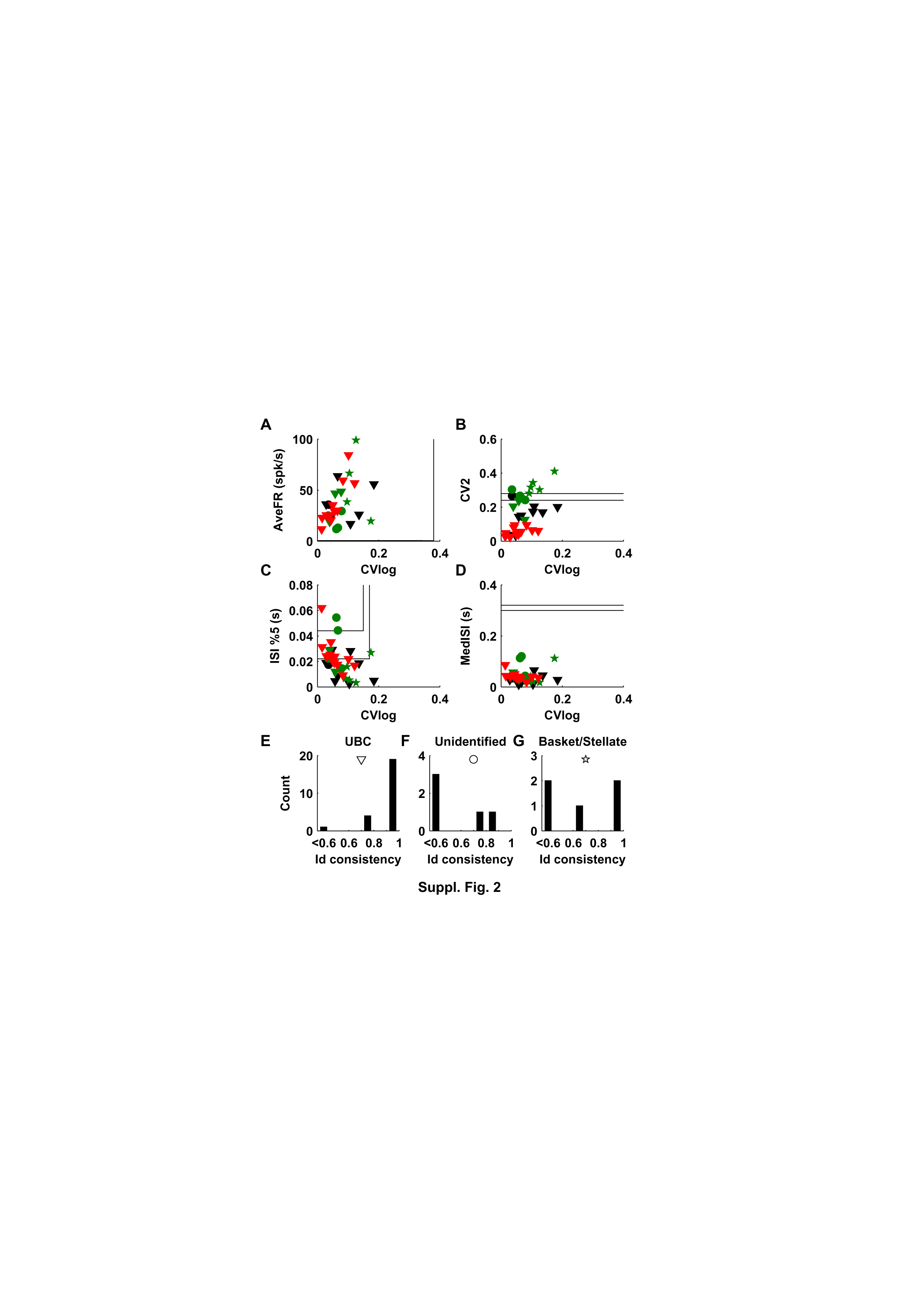

Supplement: Figure S2 — Classification of GLIs according to Ruigrok et al. (2011). (A–D) Average firing rate (A), CV2 (B), fifth percentile interval of the ISI distribution (C) and median ISI (D) as a function of the CV of the logarithm of firing frequency. Black lines represent the decision boundaries of the classification method. Circles, triangle and stars represent cells classified as ‘Unidentified’, ‘UBC’ and ‘Basket or stellate cells’. Green, red and black symbols correspond to cells which we classified as ‘Excited’, ‘Inhibited’ or ‘Undecided’. Note that this classification method follows a decision tree (see Fig. 8 in [18]). As an additional test, we investigated whether the classification method proposed by Ruigrok and colleagues [18] is sensitive to the portion of data selected for neuronal identification. Specifically, if instead of using all the spikes obtained from a given neuron for its identification we used only a few consecutive seconds of data (portions of 30 s of data, using a moving window of 30 s) our GLI population could be sorted out differently. We computed the percentage of 30 s segments for which the classification was the same as when using the entire dataset (ID consistency). Nineteen out of 24 putative UBCs (E), 0 out 5 unidentified cells (F) and 2 out of 5 cells classified as molecular layer interneurons (G) had an ID consistency of more than 90%. Therefore, altogether, only 19/34 (56%) neurons were classified consistently as a given type of GLI. Overall, it appears that the spiking activity of granular layer interneurons recorded in the ventral paraflocculus of alert macaques differs substantially from the data recorded in anesthetized rodents [18]. As a consequence, the majority of cells which were firmly identified as GLIs were not classified as such by this method. (TIF) [file pone.0082239.s002.tif]

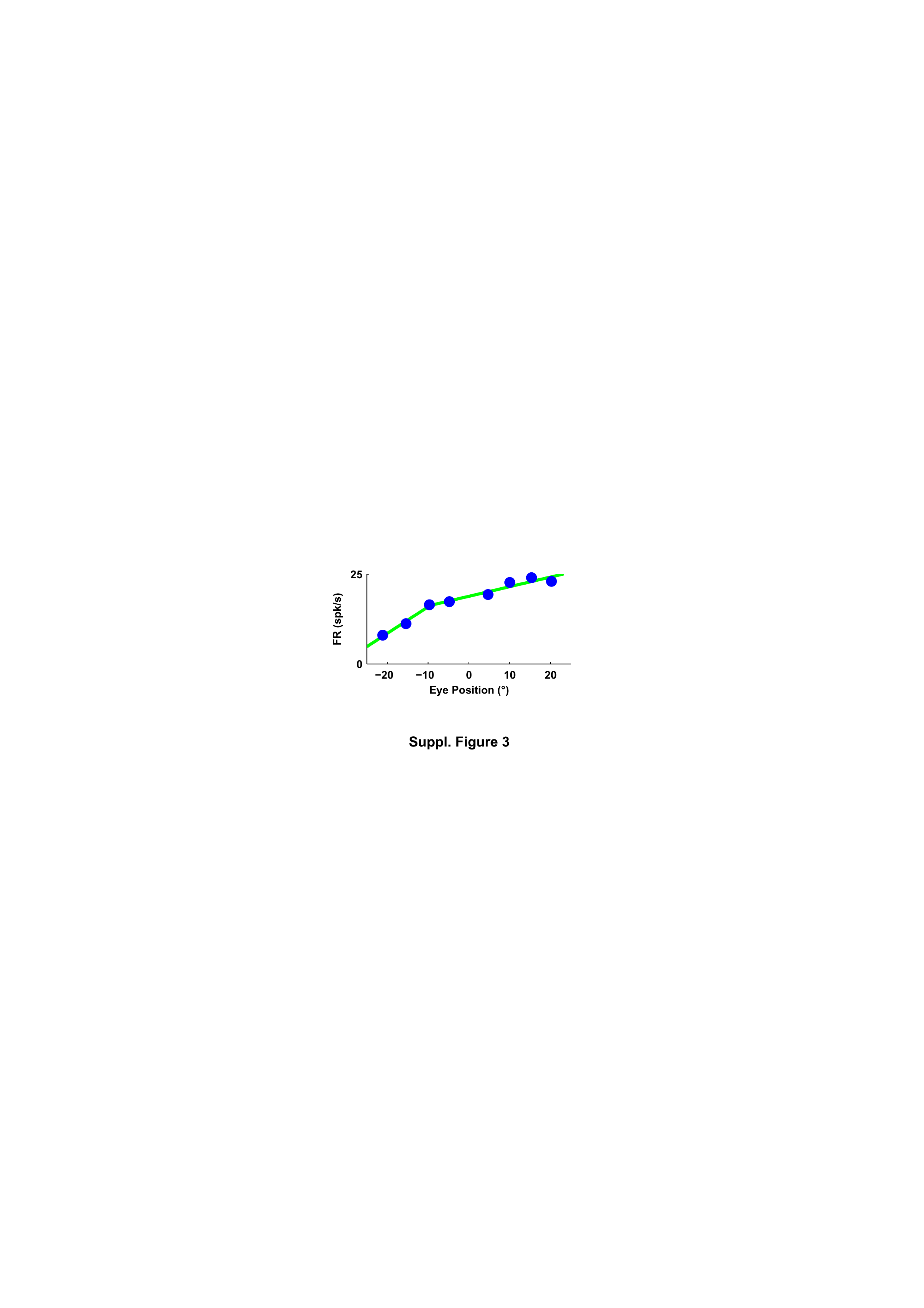

Supplement: Figure S3 — Response profile of a GLI (classified as Group 5) that showed the same directional preference for eye movements when the eye was in the right and left eye position field. All other group 4 and 5 GLIs have opposite directional preference, that is their response were best fit using two slopes of opposite sign. (TIF) [file pone.0082239.s003.tif]
