# Supplementary material for: Cerebellar Cortex Granular Layer Interneurons in the Macaque Monkey Are Functionally Driven by Mossy Fiber Pathways through Net Excitation or Inhibition
Source: PLoS One. 2013 Dec 20;8(12):e82239. doi: 10.1371/journal.pone.0082239 (PMC3869689; doi:10.1371/journal.pone.0082239)
Supplement: File S1 — (DOCX) [file pone.0082239.s004.docx]

**Supplementary Materials**

**Cerebellar cortex granular layer interneurons are functionally driven by mossy fiber pathways through net excitation or inhibition**

**Jean Laurens^1^, Heiney A. Shane^2^, GyuTae Kim^1^, Pablo M Blazquez ^1^**

1 - Department of Otolaryngology, Washington University School of Medicine, St. Louis, 63110 MO

2 – Department of Psychology, University of Pennsylvania, Philadelphia, PA 19104

Dr. Pablo Blazquez

Dept. of Otolaryngology

4566 Scott Avenue

St Louis, MO 63110-1031

Phone: 314-362-1013

Fax: 314-362-1031

Email: [pablo@pgc.wustl.edu](mailto:pablo@pgc.wustl.edu)

**Overview**

The Supplementary Material section presents details about fitting methods and data relevant to the identification and localization of Granular Layer Interneurons (GLIs). In Supplementary Figure S1, we reconstruct the sequence of molecular layer, Purkinje cell layer and granular layer encountered during a recording session. We also provide a movie (Supplementary Movie S1) in which the firing activity and spike profile of typical Purkinje cells (complex and simple spikes), mossy fibers and granular layer interneurons are shown. We show in this movie the spiking activity of a GLI (cell 5) which had a higher CV_2_ than GLIs recorded by Ruigrok and colleagues [18]. In Supplementary Figure S2, we present the results of applying Ruigrok’s classification method to our dataset.

**Functions used to classify the response profiles of GLIs**

We use one of three functions, called F_1_, F_2_ and F_3_, to fit the data. These were selected based on the variations observed in our population data. F_1_ describes neurons whose firing rate varies linearly as a function of eye position with the condition that it can’t be less than 0, i.e.:

F_1_(x) = s*x+FR_0_, if s*x+FR_0_ > 0; F_1_(x) = 0 otherwise.

In this expression, s is the slope of the linear relationship and FR_0_ the firing rate when the eye fixates straight ahead.

F_2_ describes neurons whose firing rate varies linearly only within a range of eye positions, but beyond that range the firing rate remains stable and above zero. Note that this function can be expressed by adding an offset to F_1_(x) and potentially inverting it, i.e.:

F_2_(x) = k*F_1_(x)+c, if k*F_1_(x)+c> 0; F_2_(x) = 0 otherwise.

Here, k can be equal to 1 or -1. If k takes a value of -1 it would invert the response profile of F1 function and generates a ceiling effect. We performed the fitting twice (with k = 1 and k = -1) and selected the best fit; k was not counted as a free parameter in the subsequent sequential F-test (see below).

F_3_ describes the relationship between neuronal firing rate and eye position using two lines with slopes different than zero. It is represented by the following expressions:

F_3_(x) = FR_0_-s_1_*(x_0_-x), if x < x_0_, & F_3_(x) = FR_0_ + s_2_*(x-x_0_) if x > x_0_;

F_3_ = 0 if this expression is less than 0.

Where, s_1_ and s_2_ are the slopes of the two lines, x_0_ is the eye position at which the two lines intersect and FR_0_ is the firing rate at this point. All three fitting methods account for the fact that neurons could have a recruitment threshold, thus negative firing rate values were rectified to 0.

For statistical purposes, we used another function F_0_(x) = FR_0_, which assumes that the neuron doesn't respond to eye movement. We computed the sum of squares residual SS_0_,..., SS_2_ of the fits performed with F_0_,...,F_2_ and the variance accounted for (VAF):

VAF_i_ = 1-SS_i_/SS_0_.

The best fitting function was selected by using a sequential F-test to compute the statistical significance of the increase of VAF from one fitting method (i) to another (j). We computed the following statistic:

F_i,j_ = (SS_i_-SS_j_)/(SS_j_/(N-N_j_)).

In which SS_i_ and SS_j_ are the residuals of the fits with the fitting method j and i, N_j_ is the number of parameters of the fitting method j (2, 3 and 4 for F_1_, F_2_ and F_3_ respectively) and N is the total number of data points. This statistic was compared to a Fisher distribution with N_j_-N_i_ and N-N_j_ degrees of freedom. We considered that the increase of VAF from fitting method i to j was significant if the associated p-value was less than 0.05. In addition to this criterion, we considered that the fitting method j fit the data better than i only if the increase of VAF from i to j was higher than 2%. Indeed, our aim was to select a fitting method only if it clearly fit the cell’s response better than a simpler one and to ignore small increases in VAF, even if they were significant. If no fitting method was found to be significantly better than F_0_, the cell was considered non-responsive and excluded from subsequent analysis.
